# pH-Responsive Tamoxifen-Loaded Poly(ε-caprolactone)/Poly(vinylpyrrolidone)/Poly(ethylene oxide) Fibers for Treatment of Breast Cancer: Investigation of Controlled Drug Release Properties

**DOI:** 10.1021/acsomega.6c02554

**Published:** 2026-07-02

**Authors:** Sibel Selçuk Pekdemir, Öznur Doğan Ulu, Ahmet Ulu, Mustafa Ersin Pekdemir, Ali Kuruçay, Burhan Ateş

**Affiliations:** † Polymer and Biomaterials Research Laboratory, Department of Chemistry, Faculty of Science, 37510 Fırat University, 23119 Elazığ, Turkey; ‡ Scientific and Technological Research Center, 37520 İnönü University, 44280 Malatya, Turkey; § Biochemistry and Biomaterials Research Laboratory, Department of Chemistry, Faculty of Arts and Science, İnönü University, 44280 Malatya, Turkey

## Abstract

Herein, this study reports the production of fiber mats obtained by electrospinning, produced by mixing tamoxifen (TAM) drug with polycaprolactone (PCL), polyvinylpyrrolidone (PVP), and poly­(ethylene oxide) (PEO), with the aim of eliminating cancer cells through controlled drug release. The fabricated fiber mats were thoroughly characterized by several analytical techniques. FTIR analysis indicated strong intermolecular interactions between polymer molecules of the fiber. XRD analysis revealed that fibers are semicrystalline in nature. Scanning electron microscopy verified the uniform nanofibers with bead-free morphology and the average fiber diameter ranged from 346 to 418 nm with drug loading. An increase in tensile strength (TS) and elongation at break (EAB) values of the fibers was observed after drug loading. TS and EAB was obtained up to 0.35 MPa and 88.69%, which are desired for the application. The release of TAM from the fabricated fibers strongly depended on pH value and the drug was fully released in 100 h at pH 5.5. Moreover, Higuchi, Hixson–Crowell, and Korsmeyer–Peppas kinetics confirmed the controlled release of the drug from the fiber mats via diffusion. After 24, 48, and 72 h, the cell viability rates in L929 cells were determined to be 69.7%, 88.5%, and 63.7%, respectively, while cell viability rates were calculated as 69.9%, 68.9%, and 57.7%, respectively, in MCF-7 cells. Additionally, acridine orange (AO)/ethidium bromide (EB) staining was used to detect apoptosis, and fluorescent staining images revealed apoptotic effects. Taken together, these findings suggested that the produced fiber mats could be a promising candidate for controlled TAM release in breast cancer treatment.

## Graphical abstract

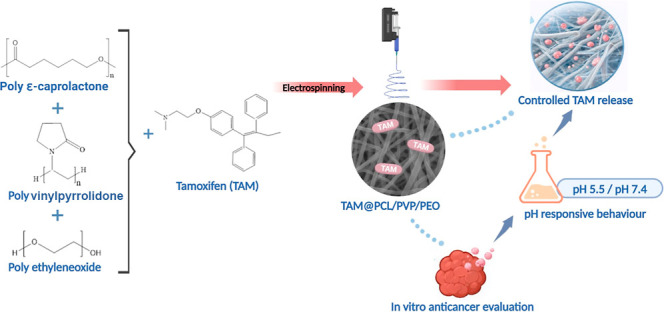

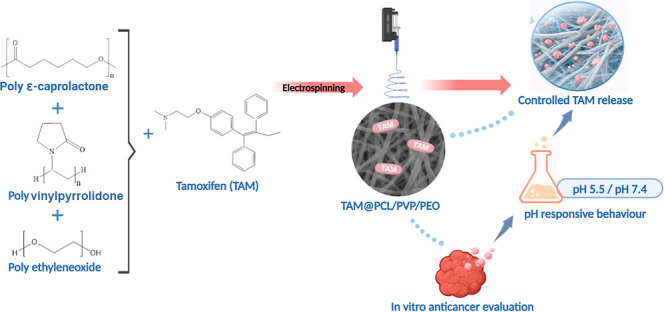

## 1 Introduction

Drug delivery systems are designed and their effects investigated according to their compatibility with the drug, target site and carrier area. There is a wide spectrum of nanomaterial forms such as nanoliposomes, nanomicelles, nanoparticles, nanocapsules, nanofibers, and nanospheres for effective drug delivery.^1^ Among them, nanofibrous structures have gained significant attention in biomedical applications due to their high surface area-to-volume ratio, porous morphology, and structural resemblance to the extracellular matrix.^2,3^ In particular, electrospun nanofibers have emerged as promising platforms for controlled drug delivery systems, offering tunable and sustained release profiles. The electrospinning technique enables homogeneous distribution of therapeutic agents within polymer matrices and supports diffusion-controlled or swelling-assisted release mechanisms.^2^ Furthermore, the high porosity and interconnected structure of nanofiber mats allow precise modulation of drug release kinetics.^3,4^

One of the most important and widely used polymers in nanofiber fabrication is polycaprolactone (PCL). PCL has several advantages, including nontoxicity, biocompatibility, biodegradability, excellent spinnability, and high tensile strength. However, it also has some disadvantages, such as hydrophobicity, low water absorption capacity, and lower cell affinity.^5^ To overcome these drawbacks, PCL is blended with many natural or synthetic polymers. For instance, poly­(vinylpyrrolidone) (PVP), a hydrophilic and biocompatible polymer, enhances water uptake capacity and improves drug solubility within polymeric matrices.^6,7^ The incorporation of PVP increases the swelling degree of nanofibers, thereby promoting controlled and potentially pH-responsive release profiles.^8,9^ Poly­(ethylene oxide) (PEO) is a semicrystalline, highly hydrophilic polymer widely used in electrospinning due to its ability to enhance chain entanglement and improve fiber formation.^10^ PEO enhances the hydrophilicity of the polymer matrix while promoting a uniform and bead-free nanofiber morphology.^6,10^ Furthermore, its strong water affinity and flexible chain structure enhance swelling behavior, and increased chain mobility in PEO-containing systems further contributes to improved drug delivery within the polymer network.^11^

Tamoxifen (TAM), a selective estrogen receptor modulator, is extensively used in the treatment of estrogen receptor-positive breast cancer.^2^ Nevertheless, its poor aqueous solubility and systemic side effects associated with conventional administration highlight the need for controlled and localized delivery strategies.^12^ At the same time, conventional systemic administration of TAM also faces several limitations, including nonspecific biodistribution, fluctuations in plasma drug levels, and limited drug accumulation in the tumor region.^13^ To overcome these limitations, various carrier matrices have been explored to enhance TAM delivery. For instance, nanoliposomes,^14^ nanomicelles,^15^ nanoparticles,^16^ nanocapsules,^17^ nanospheres,^18^ and nano/microfibers^19,20^ have been used for the efficient delivery of TAM. Among them, incorporating TAM into nanofiber-based carrier systems offers the potential to enhance its bioavailability while providing sustained and controlled release, thereby minimizing adverse effects.^21,22^ According to several studies, the electrospun fibers are excellent materials for the delivery and release of TAM. For example, Liu et al.^19^ suggested silk fibroin/2-hydroxypropyl-β-cyclodextrin composite fibers as a matrix for the local TAM delivery during cancer therapy. In another work, Nie et al.^20^ fabricated the polyacrylonitrile fibers loaded with TAM citrate. Pinzón-García et al.^21^ produced PCL nanofibers as an adjuvant strategy for TAM release. In a recent study, Li et al.^22^ reported the trichamber nanofibers for the sustained release of TAM citrate. However, in the previous literature, a limited number of electrospun fiber mats have been reported for the release of TAM. Therefore, TAM was used as a model drug to evaluate the drug release profile and kinetics in this study.

Although TAM-loaded polymeric delivery systems have been reported to date, studies investigating pH-sensitive electrospun fiber mats for localized TAM delivery have been limited. Furthermore, the physicochemical properties, intermolecular interactions, and pH-dependent release characteristics of TAM using hydrophobic PCL in combination with hydrophilic PVP and PEO have not been sufficiently investigated in the literature. To our knowledge, PCL/PVP/PEO fibers mats were not previously reported in the loading and controlled release of TAM. The developed TAM-loaded electrospun PCL/PVP/PEO fiber mats were designed as a macroscopic local drug delivery platform rather than a systemic nanocarrier system. Electrospun nanofiber mats are particularly attractive for this purpose due to their high surface area-to-volume ratio, tunable porosity, extracellular matrix-like architecture, and controlled release behavior, as well as their ability to integrate hydrophobic anticancer drugs.^23^ The aim of the proposed system was to provide localized and sustained TAM delivery to breast tissue. Compared to conventional systemic TAM application, local nanofiber delivery systems can provide several advantages, including sustained and site-specific drug release, reduced systemic exposure, and lower side effects. Therefore, this innovative research aimed to overcome the known limitations of TAM drug according to the current literature, while also improving the loading potential, on-demand release, and therapeutic efficacy of TAM. For this purpose, pH-sensitive TAM-loaded PCL/PVP/PEO fibers mats were fabricated through a one-step electrospinning method and we assessed the chemical, thermal, morphological characterization and crystalline properties of the fabricated fiber mats. Then, the effect of the pH medium was investigated on the release of TAM. More specifically, the release rate of TAM was significantly higher under pH 5.5 compared to under pH 7.4. Furthermore, the different kinetic models, including zero-order, first-order, Higuchi, Hixson–Crowell, Korsmeyer–Peppas employed to understand a deeper release mechanistic. Finally, and most importantly, we investigated the cytotoxicity of TAM-loaded PCL/PVP/PEO fibers mats on both cancerous (MCF-7) and normal (L929) cell lines and cell death was revealed to indicate apoptotic effects using fluorescent staining. This investigation was designed to improve the stability and bioavailability of TAM and to make it a promising candidate in drug delivery systems.

## 2 Experimental Section

### 2.1 Materials

PCL (*M*_n_ ≈ 80,000 g/mol) was purchased from Sigma-Aldrich. PVP (*M*_v_ ≈ 10,000 g/mol) was obtained from abcr GmbH (Karlsruhe, Germany), and PEO (*M*_v_ ≈ 200,000 g/mol) was supplied by Sigma-Aldrich. TAM was purchased from Carbosynth (Compton, UK). Chloroform (absolute grade) was obtained from Carlo Erba Reagents (Val-de-Reuil, France). Methanol (≥99.7%, GC grade) was supplied by Honeywell (Charlotte, NC, USA). All other chemicals and reagents used were of analytical purity and were used without any further purification. The water used in all experiments was produced via a New Human Power I S-UV water purification system (Human Corporation, Seoul, South Korea).

### 2.2 Electrospinning Solutions

Initially, 1.2 g of PCL was dissolved in a 3:1:1 (v/v/v) solvent mixture containing *N*,*N*-dimethylformamide (DMF), chloroform, and methanol. To ensure complete dissolution of PCL, the mixture was continuously stirred on a magnetic stirrer at room temperature for approximately 2 h. The resulting clear PCL solution was then mixed with 0.12 g of PVP. To ensure homogeneous distribution and complete dissolution of PVP, the system was stirred continuously on a magnetic stirrer for an additional 1–2 h. Finally, 0.048 g of PEO was added to the mixture. To ensure complete dissolution of the polymer components and obtain a homogeneous mixture, the prepared solution was stirred on a magnetic stirrer at room temperature (25 ± 2 °C) for 24 h.

### 2.3 Fabrication of TAM-Loaded Fibers

The electrospinning process was employed to fabricate the PCL/PVP/PEO and TAM-loaded PCL/PVP/PEO fibers mats. For the fabrication of drug-loaded fibers, TAM corresponding to 4 wt % of the total polymer content (54.7 mg with respect to 1.368 g total polymer) was first dissolved in methanol to obtain a clear drug solution. TAM solution was then added dropwise into the previously prepared polymer solution under continuous stirring and further stirred for an additional 2 h to ensure homogeneous distribution of the drug within the spinning solution. Electrospinning was carried out using an electrospinning/spraying system (FYTRONIX 9000 electrospin system, Türkiye). The prepared polymer solution was loaded into a 2.5 mL plastic syringe equipped with a 21 G stainless-steel needle (0.80 × 38 mm) and electrospun at an applied voltage of 15 kV, a solution flow rate of 1.0 mL h^–1^, and a tip-to-collector distance of 20 cm. The fiber mats were collected on aluminum foil under ambient conditions. After electrospinning, the as-spun fiber mats were dried in a vacuum oven at 50 °C to remove residual solvents and stored for further characterization and experiments.

### 2.4 Characterization Methods

Fourier transform infrared spectroscopy (FTIR, Spectrum Two, PerkinElmer, UK) equipped with attenuated total reflection was used to characterize the binding configurations of with and without drug mats produced by the electrospinning method. FTIR spectra were recorded at a resolution of 4 cm^–1^ in the range 4000 to 400 cm^–1^. The surface morphology of the fiber mats was examined using a scanning electron microscope (SEM, LEO-EVO 40, Cambridge, England). All samples were coated with a 40:60 gold–palladium alloy. In addition, the fiber diameter of randomly selected mats was determined by analyzing SEM images using ImageJ 1.54g software (NIH, USA). Incorporation of TAM was also ascertained by energy-dispersive X-ray spectroscopy (EDS). XRD analysis of the fibers was performed using an X-ray diffractometer (Rigaku RadB-Dmax II model, USA) with Cu Kα (λ = 1.54 A°) radiation at 40 kV and 40 mA. The range of diffraction angle 2θ was 5–80° using a scanning speed of 6°/min. The data was graphed using OriginPro 2021 software. The mechanical properties of the fibers were evaluated by tensile tests before and after TAM loading. Briefly, the fiber mats were cut to dimensions of 100 mm × 5 mm (length × width) and the average thickness of the fiber mats was measured using a digital caliper (MAGTOTO, measurement range: 0–25 mm and 0.001 mm accuracy) at three different locations. Mechanical tests were performed on an electronic tensile testing machine with a 5 kN load cell (MTS Exceed 40 series, USA). Tests were conducted at room temperature (25 ± 1 °C) and humidity conditions (45–55% relative humidity) at a speed of 0.847 mm per second and a data acquisition rate of 10.0 Hz. Three replicates were used for each mat.

### 2.5 Swelling Degree and Water Uptake Measurements

The swelling behavior and water uptake capacity of the fiber mats were evaluated using a gravimetric method. The samples were first dried to constant weight, and their dry mass (*W*_d_) was recorded. Both PCL/PVP/PEO and TAM-loaded PCL/PVP/PEO fiber mats were then immersed in phosphate-buffered saline (PBS) solutions adjusted to pH 7.4 and pH 5.5 and incubated at 37 °C. At predetermined time intervals (15, 30, 45, and 60 min), the samples were withdrawn from the medium, gently blotted with soft filter paper to remove excess surface liquid, and immediately weighed to obtain the swollen mass (*W*_s_). The measurements were continued until equilibrium swelling was achieved. For water uptake determination, the swollen samples were subsequently dried to constant weight, and the final dry mass (*W*_d_) was recorded. The swelling degree and water uptake were calculated according to eqs 1 and 2, respectively.

1 $$Swelling\;ratio\;\left(\%\right)=\frac{W_{s}-W_{d}}{W_{d}}\times 100$$

2 $$Water\;uptake\;\left(\%\right)=\frac{W_{s}-W_{d}}{W_{s}}\times 100$$

### 2.6 *In Vitro* Hydrolytic Degradation

The *in vitro* hydrolytic degradation of the fiber mats was evaluated following the ISO 10993-9 guideline. The samples were cut into pieces of approximately 2 × 2 cm^2^, dried to constant weight, and their initial mass (*W*_i_) was recorded. Both drug-free and TAM-loaded PCL/PVP/PEO nanofibers were immersed in phosphate-buffered saline (PBS) solutions adjusted to pH 7.4 and pH 5.5 and incubated at 37 °C for up to 28 days. At predetermined time intervals, the samples were withdrawn from the medium, dried at 70 °C to constant weight, and then weighed again to obtain the final mass (*W*_f_). The hydrolytic degradation degree of the membranes was determined based on the weight loss and expressed as percentage weight loss according to eq 3.

3 $$Weight\;loss\;\left(\%\right)=\frac{W_{i}-W_{f}}{W_{i}}\times 100$$

### 2.7 *In Vitro* Drug Release

To determine the release of the drug TAM from fiber mats, experiments were performed at 37 °C in PBS pH 5.5 and 7.4, simulating the tumor and physiological pH microenvironments, respectively. Briefly, approximately 26 mg of fibers (0.1905 mg TAM/cm^2^ fiber) was cut into specimens (20 × 20 mm) and were immersed in 3 mL PBS.^21^ Due to the solubility limitation of TAM in aqueous solutions, the releasing medium contained 30% ethanol was used.^20^ The samples were then incubated at a shaking chamber at 37 °C with a speed of 100 rpm. At predetermined time points, 1.5 mL of release solution was taken from the release medium and immediately replaced with fresh PBS of the same volume, pH, and temperature. The absorbance of the release solution was measured at 280 nm using a UV–vis spectrophotometer and the amount of released drug was determined using TAM calibration curve (*R* ^2^ = 0.9999). The release processes were repeated using three independent samples and the results were expressed as time-dependent cumulative release.

### 2.8 Release Kinetic Analysis

To investigate the kinetics of TAM release, data were adapted to the five distinct kinetic models, including zero-order, first-order, Higuchi, Hixson–Crowell, and Korsmeyer–Peppas. The best-fitted model was determined by the correlation coefficient. Mathematical equations of each model were expressed in the following eqs 4–8 ^24,25^

4 $$Q_{t}=k_{o}\cdot t,zero‐order$$

where *Q*_*t*_ is the quantity of TAM dissolved in the time *t* and *k*_o_ is the zero-order release constant.

5 $$\log Q_{t}=k\cdot t/2.303,first‐order$$

where *Q*_*t*_ is the amount of TAM released in the time *t* and *k*_1_ is the first order release constant.

6 $$Q_{t}=k_{H}\cdot t^{1/2},Higuchi$$

where *Q*_*t*_ is the amount of TAM released in the time *t* and *k*_H_ is the Higuchi dissolution constant.

7 $${W_{o}}^{1/3}-{W_{t}}^{1/3}=k\cdot t,Hixson-Crowell$$

where *W*_o_ is the initial amount of TAM in formulations, *W*_*t*_ is the remaining amount of TAM in formulation at time *t* and *k*_HC_ is a constant.

8 $$M_{t}/M_{\infty }=k\cdot t^{n},Korsmeyer-Peppas$$

where *M*_*t*_/*M*_∞_ is the fraction of TAM released at the time *t*, *k*_P_ is the release rate constant and *n* is the release exponent.

### 2.9 Biocompatibility and *In Vitro* Anticancer Activity

From each electrospun fiber material, 0.05 g was cut and transferred into the same sterile tube containing 1 mL of high glucose DMEM (Gibco) supplemented with 10% FBS (Capricorn Scientific) and 1% Pen–Strep (Capricorn Scientific). The samples were incubated at 37 °C for 3 days in a 5% CO_2_ incubator (Panasonic MCO-18AC). After incubation, the fiber materials were removed from the medium and the conditioned DMEM was collected. Mouse fibroblast cells (L-929) and human breast cancer cells (MCF-7) were seeded separately into transparent 96-well plates (Nest) at a density of 10,000 cells per well and allowed to adhere in a CO_2_ incubator. After cell adhesion, the culture medium was removed and 100 μL of fiber-conditioned DMEM was added to each well. The plates were incubated for 24, 48, and 72 h. Prior to the MTT assay, cell images were captured under visible light using an inverted microscope (Olympus CKX41) equipped with an integrated camera (Olympus DP74). After imaging, the medium was removed and replaced with DMEM containing 10% MTT solution. The MTT (BLDpharm) solution was prepared in PBS at a concentration of 5 mg/mL and adjusted to pH 7.4. The plates were incubated for 4 h. Following incubation, the MTT-containing medium was aspirated and 100 μL of DMSO (Sigma-Aldrich) was added to each well to dissolve the formazan crystals completely. After ensuring complete dissolution, the absorbance of each well was measured at 540 nm using a spectrophotometer (BioTek Synergy LX).

### 2.10 Fluorescence Microscopy

The apoptotic effects of the control, PCL/PVP/PEO, and PCL/PVP/PEO/TAM groups were evaluated in MCF-7 and L929 cell lines using the acridine orange (AO)/ethidium bromide (EtBr) double fluorescence staining method. Cells were stained with a mixture of AO and EtBr after 24, 48, and 72 h of incubation. Fluorescent images were obtained using fluorescence microscopy (Olympus CKX41) with fluorescein isothiocyanate (FITC) and propidium iodide (PI) filters, and the image channels were combined for analysis. Red and green fluorescence intensities were calculated using ImageJ software (version 1.54s), and apoptotic signal intensities were evaluated.

## 3 Results and Discussion

### 3.1 FTIR Analysis

Figure 1A presents the ATR-FTIR spectra of pristine PCL, PVP, and PEO, while Figure 1B shows the spectra of PCL/PVP/PEO and TAM-loaded PCL/PVP/PEO fiber mats. The spectra of pristine polymers were initially examined to identify their characteristic absorption bands. In the spectrum of pristine PVP, the broad band around 3410 cm^–1^ was attributed to O–H stretching vibrations, whereas the bands at 2930–2860 cm^–1^ corresponded to asymmetric and symmetric −CH_2_ stretching vibrations.^26–28^ The characteristic carbonyl (C=O) stretching vibration of PVP was observed at 1654 cm^–1^ together with several C–H and C–N-related bands.^29–33^ For pristine PEO, the characteristic −CH_2_ stretching vibration appeared at 2878 cm^–1^, while the prominent C–O–C stretching vibration was detected at approximately 1097 cm^–1^.^34–38^ In the ATR-FTIR spectrum of pristine PCL, the characteristic ester carbonyl (C=O) stretching vibration was observed at around 1720 cm^–1^, accompanied by typical −CH_2_ and C–O–C-related absorption bands.^39–42^ The characteristic absorption bands of TAM were identified at 3025 cm^–1^ (O–H stretching), 1605–1510 cm^–1^ (aromatic C=C stretching), and within the 1200–900 cm^–1^ region corresponding to C–N and C–O-related vibrations.^43–46^ As shown in Figure 1B, the ATR-FTIR spectrum of the PCL/PVP/PEO fibers exhibited the characteristic absorption bands of all constituent polymers, confirming the successful formation of the composite nanofibrous structure without altering the chemical backbones of the polymers. No new absorption bands were observed after blending, indicating the absence of chemical modification between the polymer components. Similarly, the TAM-loaded PCL/PVP/PEO fibers retained the main characteristic bands of the polymer matrix after TAM incorporation. Slight intensity variations and weak shoulders observed in the regions of 1600–1510 cm^–1^ and 1200–900 cm^–1^ further supported the presence of TAM within the nanofiber matrix. However, the characteristic absorption bands of TAM were not distinctly resolved because of its relatively low loading content and overlap with the intense polymer bands, which is commonly reported in drug-loaded electrospun nanofibrous systems.^47–49^ These findings indicated that TAM was successfully incorporated into the PCL/PVP/PEO fiber matrix without inducing chemical modification of the polymeric components. Scheme 1 shows schematic illustration of the possible interactions between TAM and PCL/PVP/PEO polymers within the electrospun fiber matrix. Hydrophobic interactions between the aromatic moieties of TAM and the hydrophobic PCL chains likely contributed to the incorporation of the drug into the polymeric matrix. Dipole–dipole interactions may occur between the carbonyl groups of PVP and the polar functional groups of TAM, which could support the homogeneous distribution of the drug within the fiber structure. Additionally, weak polar interactions between PEO chains and TAM may contribute to intermolecular compatibility within the polymer blend. In addition, followed SEM and XRD measurements confirmed this hypothesis.

**Figure 1:**
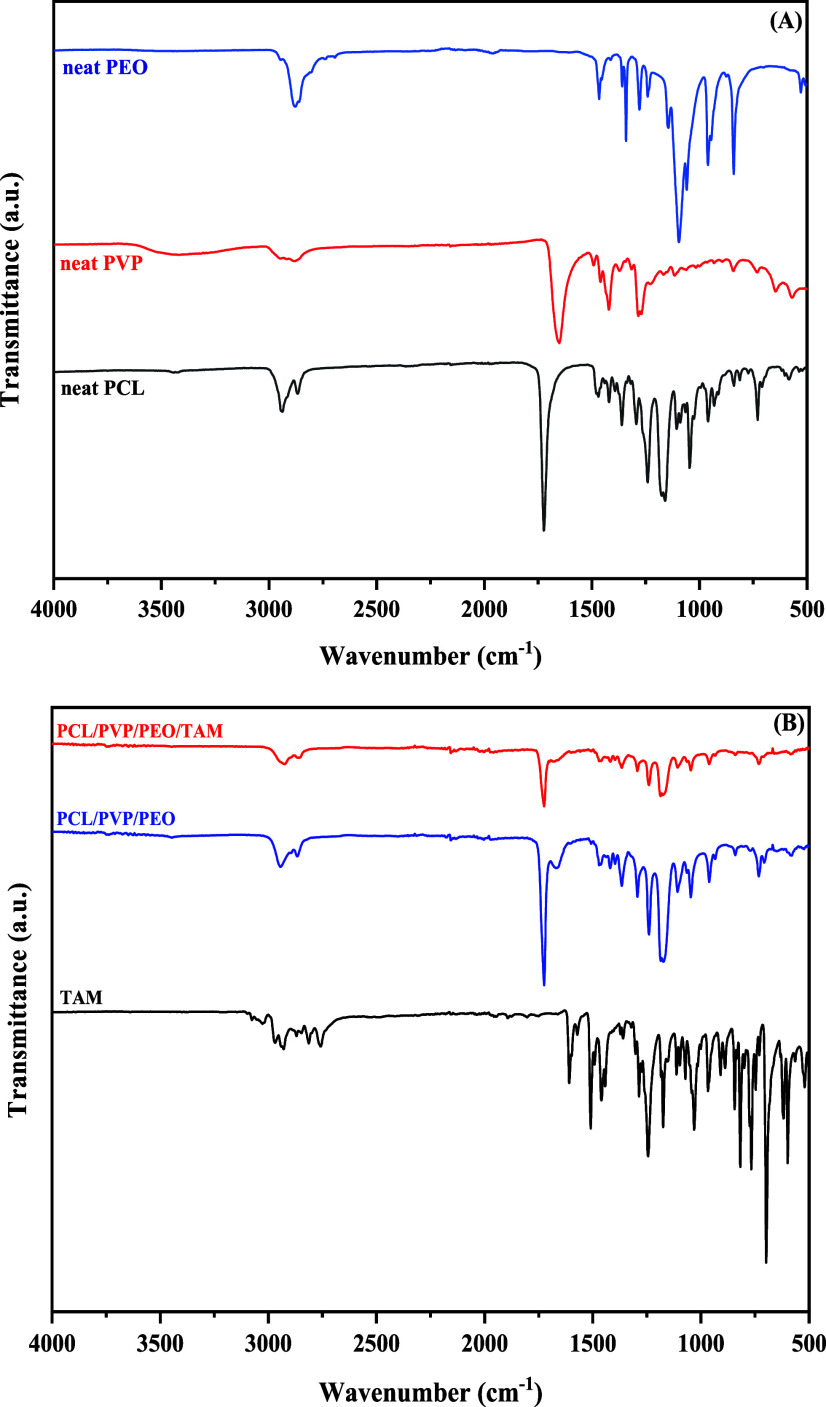
ATR-FTIR spectra of neat PCL, PVP, and PEO (A), drug-free and TAM loaded PCL/PVP/PEO ternary blend fibers (B).

**Scheme 1:**
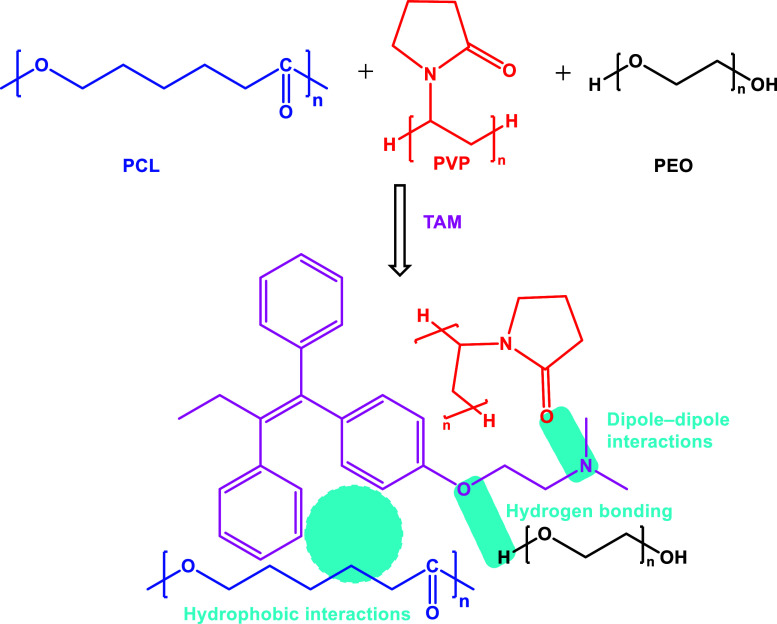
Schematic Illustration of the Possible Interactions between TAM and PCL/PVP/PEO Polymers within the Electrospun Fiber Matrix

### 3.2 Surface Morphology

The surface morphology of the PCL/PVP/PEO and TAM-loaded PCL/PVP/PEO fibers was examined using SEM, with the results presented in Figure 2. SEM images clearly illustrated that pH-sensitive fiber mats prepared with and without TAM exhibited a network structure consisting of continuous, randomly oriented, and rod-shaped fibers without any beads. In addition, all fibers have a mean diameter that falls within the microscale range. None of the fibers contain beads or any defects within the fiber network. As observed, the surface of the fibers was smooth, and no drug crystals were observed on the fiber surface. Therefore, TAM molecules were well integrated into the fibers produced by the electrospinning method. Moreover, average fiber diameter and distribution were calculated by analyzing SEM images using ImageJ 1.54g software. The average diameters of PCL/PVP/PEO and TAM-loaded PCL/PVP/PEO fiber mats were found to be 346 ± 60 nm and 418 ± 43 nm, respectively. In another words, the incorporation of TAM drug into the fibers increased the diameter of fibers slightly (Figure 3). However, this increase in average fiber diameter was not a statistically significant difference. Previous literature has reported that adding drugs to the polymer solution increased the fiber diameter.^50–52^ Additionally, the observed fiber diameter range (346–418 nm) can be considered beneficial for localized TAM delivery applications. Fibers within this size range may facilitate efficient drug loading and controlled pH-sensitive release behavior by providing a high interfacial area. Furthermore, these fibers may potentially enhance cell–material interactions at the implantation site by mimicking the structural features of the extracellular matrix.^6^

**Figure 2:**
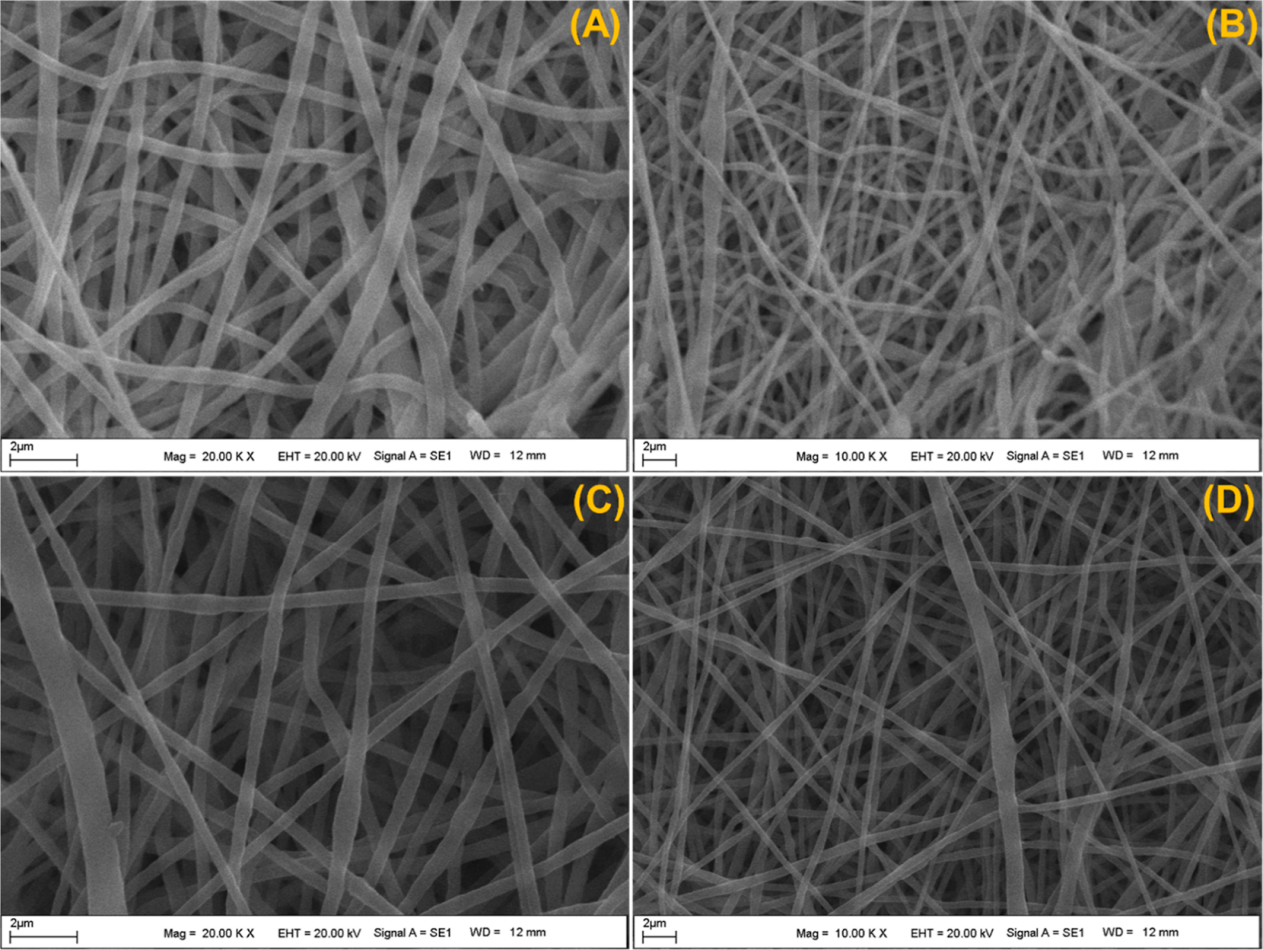
SEM images of PCL/PVP/PEO (A,B) and TAM-loaded PCL/PVP/PEO fibers (C,D).

**Figure 3:**
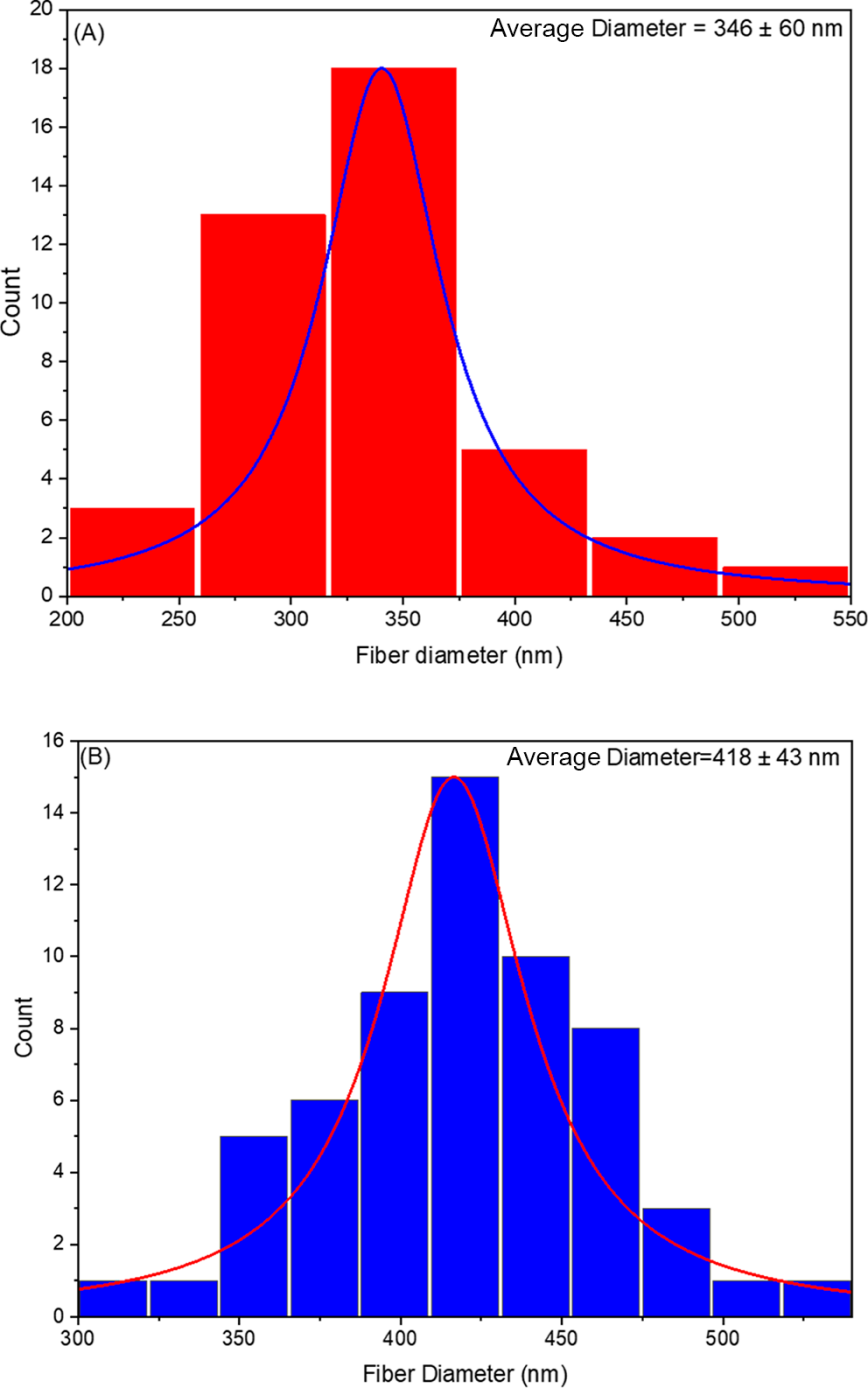
Fiber diameter distribution of PCL/PVP/PEO (A) and TAM-loaded PCL/PVP/PEO fiber mats (B).

Furthermore, EDS patterns of carbon (C), nitrogen (N), and oxygen (O) elements demonstrated the uniform distribution and adequate interactions of polymers and drug within the fibers (Figure 4A,B). As observed in the EDS results, TAM-loaded PCL/PVP/PEO fiber showed a higher nitrogen content and a higher oxygen content compared to the PCL/PVP/PEO fiber. This phenomenon proved the accuracy of TAM loading onto the fibers.

**Figure 4:**
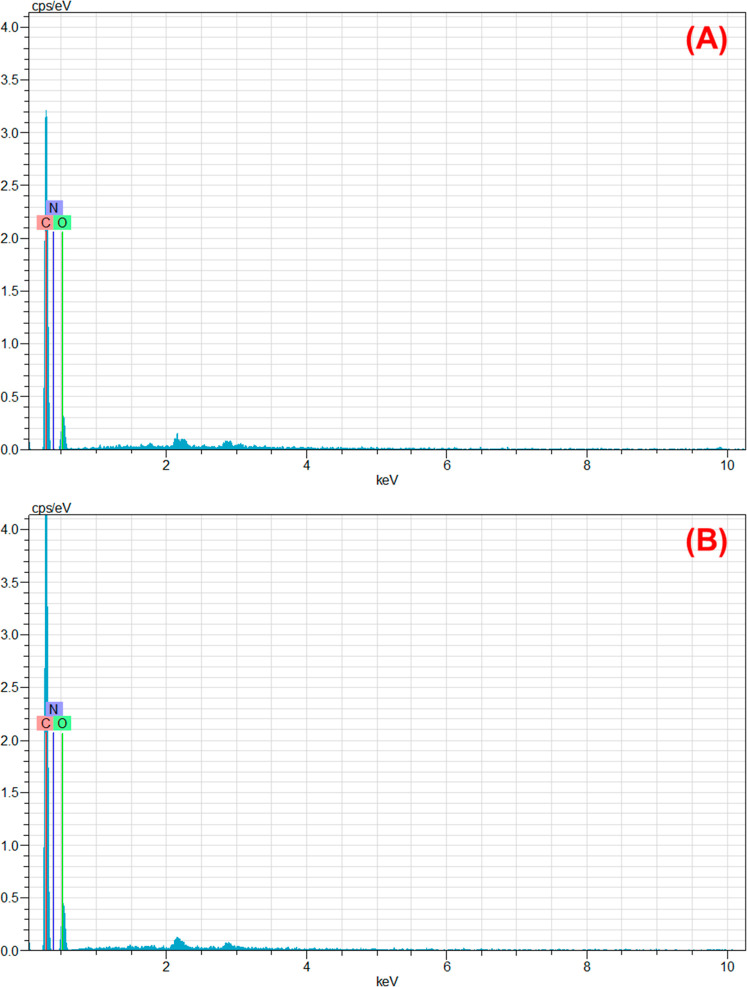
EDS spectra of the PCL/PVP/PEO (A) and TAM-loaded PCL/PVP/PEO fibers (B).

### 3.3 XRD Analysis

XRD analysis was carried out on PCL/PVP/PEO and TAM-loaded PCL/PVP/PEO fibers as shown in Figure 5. The narrow peaks at 2θ = 21.14° and 2θ = 23.68° were assigned to the semicrystalline structure of PCL, corresponding to the (110) and (200) crystal planes, respectively.^53–55^ This confirmed that PCL was the main component in the polymer mixture. Due to the low content and embedded morphology of PVP and PEO polymers in the composite fiber, it was difficult to distinguish the typical peaks of these polymers. The absence of an observed signal was an expected outcome. Furthermore, the amorphous nature of PVP and PEO did not alter the position of the sharp crystalline peaks of PCL. This indicated that the PCL crystal lattice was preserved within the mixture and that the secondary polymers have a limited effect on the crystal structure. However, the presence of PVP and PEO was supported by the FTIR spectrum of the fibers. On the other hand, in TAM-loaded PCL/PVP/PEO fibers, the reduction in the intensity of the characteristic PCL peaks, while maintaining their positions, revealed that the drug and low-ratio amorphous polymers partially hinder the regular packing of PCL chains, leading to a decrease in the degree of crystallization. However, the absence of sharp crystalline peaks specific to TAM indicated that the drug was dispersed at an amorphous or molecular level within the polymer matrix and did not form a separate crystalline phase.

**Figure 5:**
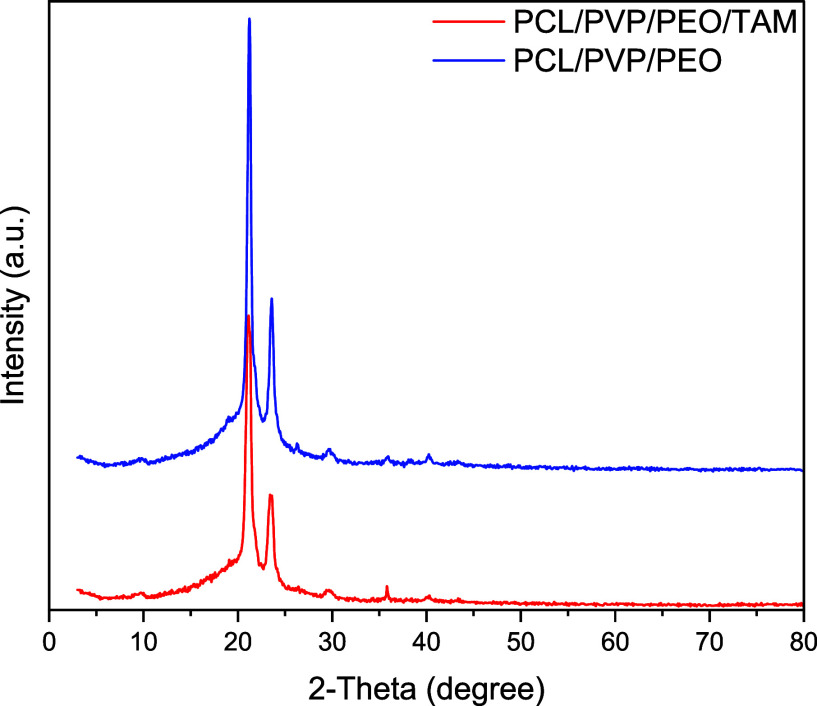
XRD patterns of PCL/PVP/PEO and TAM-loaded PCL/PVP/PEO fibers.

### 3.4 TGA Analysis

Figure 6 shows the thermogravimetric mass loss curves of PCL/PVP/PEO and TAM-loaded PCL/PVP/PEO fibers recorded under increasing temperature. The TGA curve of the PCL/PVP/PEO fibers exhibited a single-step degradation behavior, with the onset degradation temperature (*T*_i_) observed at approximately 360 °C. In contrast, the TGA curve of TAM-loaded PCL/PVP/PEO fibers showed a decrease in *T*_i_ to around 240 °C. This reduction in *T*_i_ can be attributed to the relatively low thermal stability of TAM itself, as its thermal degradation temperature and maximum decomposition temperature have been reported to be around ∼280 °C in the literature.^56,57^ Moreover, the PCL/PVP/PEO fibers exhibited a residual mass of approximately 5.05% at 500 °C, whereas TAM-loaded PCL/PVP/PEO fibers showed a lower residue of about 2.91%. Drug incorporation is known to increase the free volume within the polymer matrix and to introduce thermally less stable domains, which act as preferential degradation sites, leading to an earlier onset of thermal decomposition and a reduced char/residual yield.^58–60^ Overall, the observed decrease in both *T*_i_ and residual mass upon TAM loading indicates a slight reduction in the thermal stability of the PCL/PVP/PEO fiber system.

**Figure 6:**
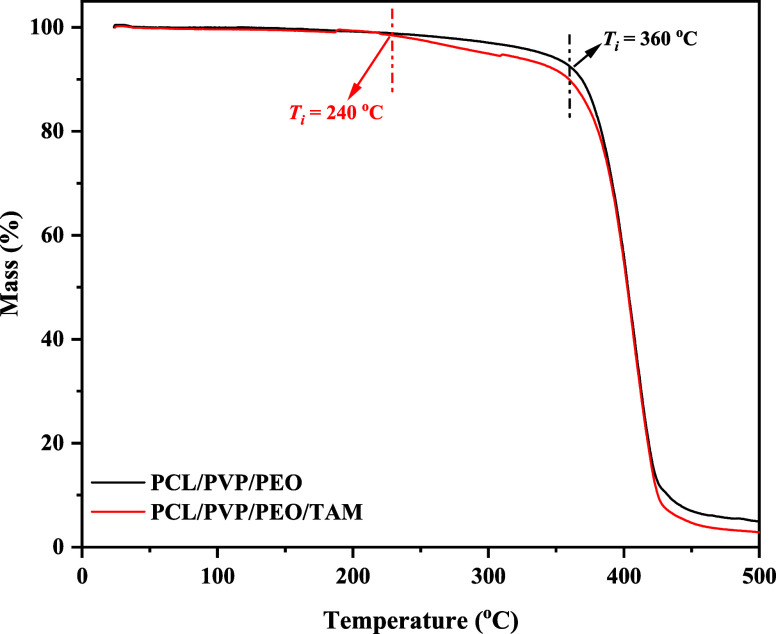
TGA curves of PCL/PVP/PEO and TAM-loaded PCL/PVP/PEO fiber mats recorded under a nitrogen atmosphere at a heating rate of 10 °C min^–1^.

### 3.5 Mechanical Property Test

Investigating the tensile properties of drug-loaded fiber mats is extremely important. Henceforth, the mechanical properties of the fiber mats were investigated. The stress–strain curves of PCL/PVP/PEO and TAM-loaded PCL/PVP/PEO fibers are shown in Figure 7A. Mechanical parameters such as TS and EAB values of the fabricated fibers are also shown in Figure 7B. TAM addition significantly reduced the brittleness of PCL/PVP/PEO fibers and improved its mechanical properties. The addition of TAM to the fiber mats increased the TS of PCL/PVP/PEO fibers from 0.32 ± 0.06 MPa to 0.35 ± 0.03 MPa. As shown in Figure 7B, the EAB values of PCL/PVP/PEO and TAM-loaded PCL/PVP/PEO fibers were 78.57 ± 10.50% and 88.69 ± 0.91%, respectively. Our study showed that the addition of TAM not only increased the tensile strength of PCL/PVP/PEO fibers but also increased its flexibility. The increase in TS and EAB values revealed that interactions have occurred between the polymer matrix and TAM and these interactions improved the free movement of the polymer chains. Other researchers also reported that drug loading improved the tensile strength of the carrier polymer.^61,62^ In conclusion, the mechanical properties of the fabricated fibers have also confirmed their applicability in the drug delivery process.

**Figure 7:**
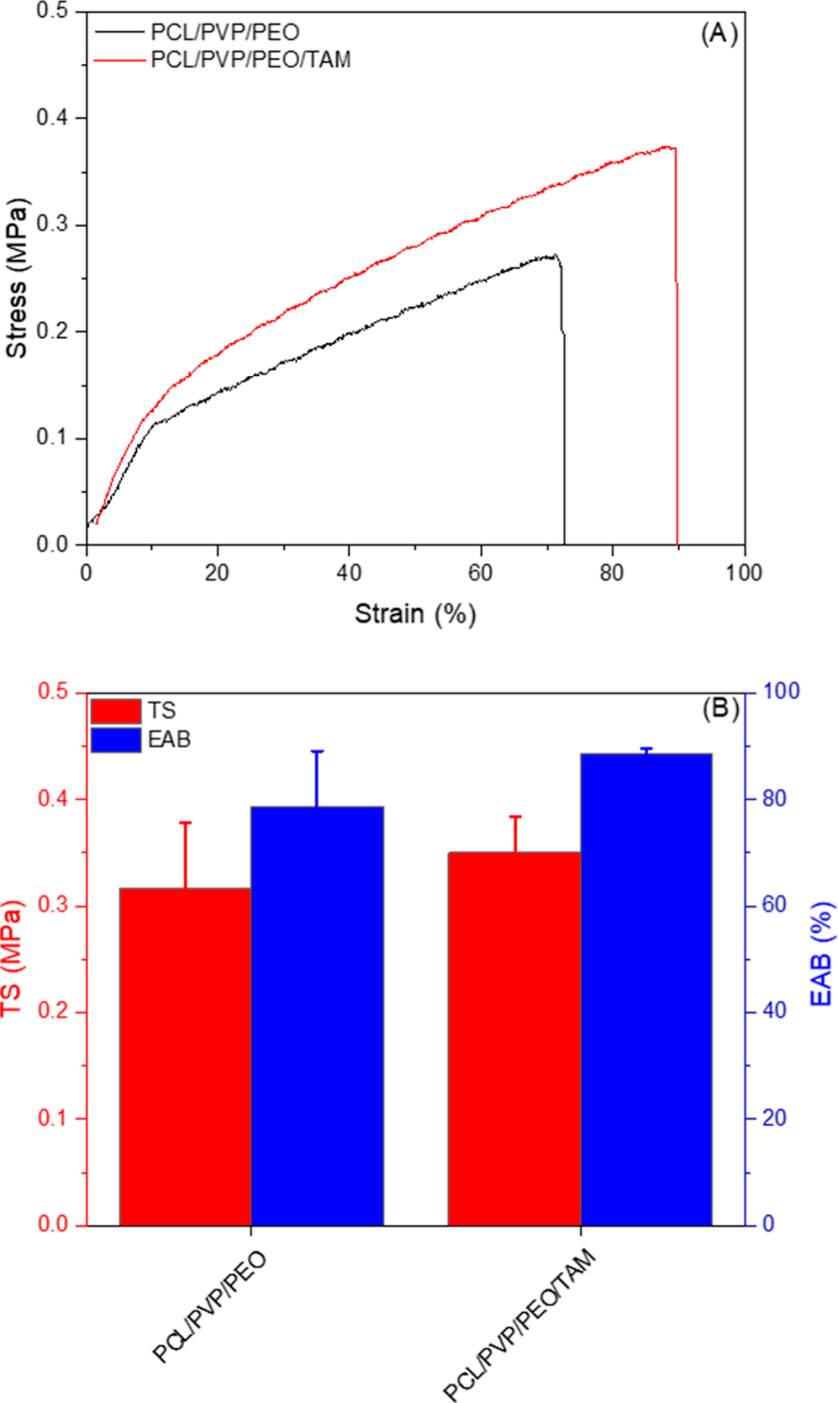
Stress–strain curves (A) and mechanical properties of PCL/PVP/PEO and TAM-loaded PCL/PVP/PEO fibers (B).

### 3.6 Swelling Degree and Water Uptake Behavior

The swelling behavior of PCL/PVP/PEO and TAM-loaded PCL/PVP/PEO fiber mats was evaluated as a function of time under pH 5.5 and pH 7.4 conditions (Figure 8A). All sample groups exhibited a progressive increase in swelling degree over time, indicating continuous hydration of the polymeric network. This time-dependent swelling trend is frequently observed in hydrophilic polymer blends due to rapid water ingress and polymer chain relaxation.^63,64^ At 15 min, the swelling degree of PCL/PVP/PEO at pH 5.5 was 491 ± 3.4%, which increased to 524 ± 4.8% by 75 min. A similar trend was observed at pH 7.4 for the PCL/PVP/PEO, with swelling increasing from 498 ± 4.6% to 559 ± 4.9%. For TAM-loaded fiber mats, the swelling degree at pH 5.5 increased from 553 ± 4.2% at 15 min to 614 ± 4.7% at 75 min, while at pH 7.4 it rose from 607 ± 3.5% to 710 ± 4.3%. These results clearly indicate that the presence of TAM enhanced the overall swelling degree compared with drug-free membranes, which may be attributed to changes in microstructure and increased free volume facilitating water penetration.^65,66^ This enhanced swelling behavior has also been reported in other drug-loaded fiber systems with hydrophilic components.^6,67^ A consistent pH effect was observed, with membranes exhibiting higher swelling degrees at near-physiological pH 7.4 than at acidic pH 5.5 at all time points (e.g., 710 ± 4.3% vs 614 ± 4.7% for TAM-loaded PCL/PVP/PEO fibers at 75 min), which is consistent with literature reports indicating that hydrophilic polymer networks swell more extensively at neutral to slightly basic pH due to enhanced ion–dipole interactions and reduced polymer–water interfacial tension.^68,69^

**Figure 8:**
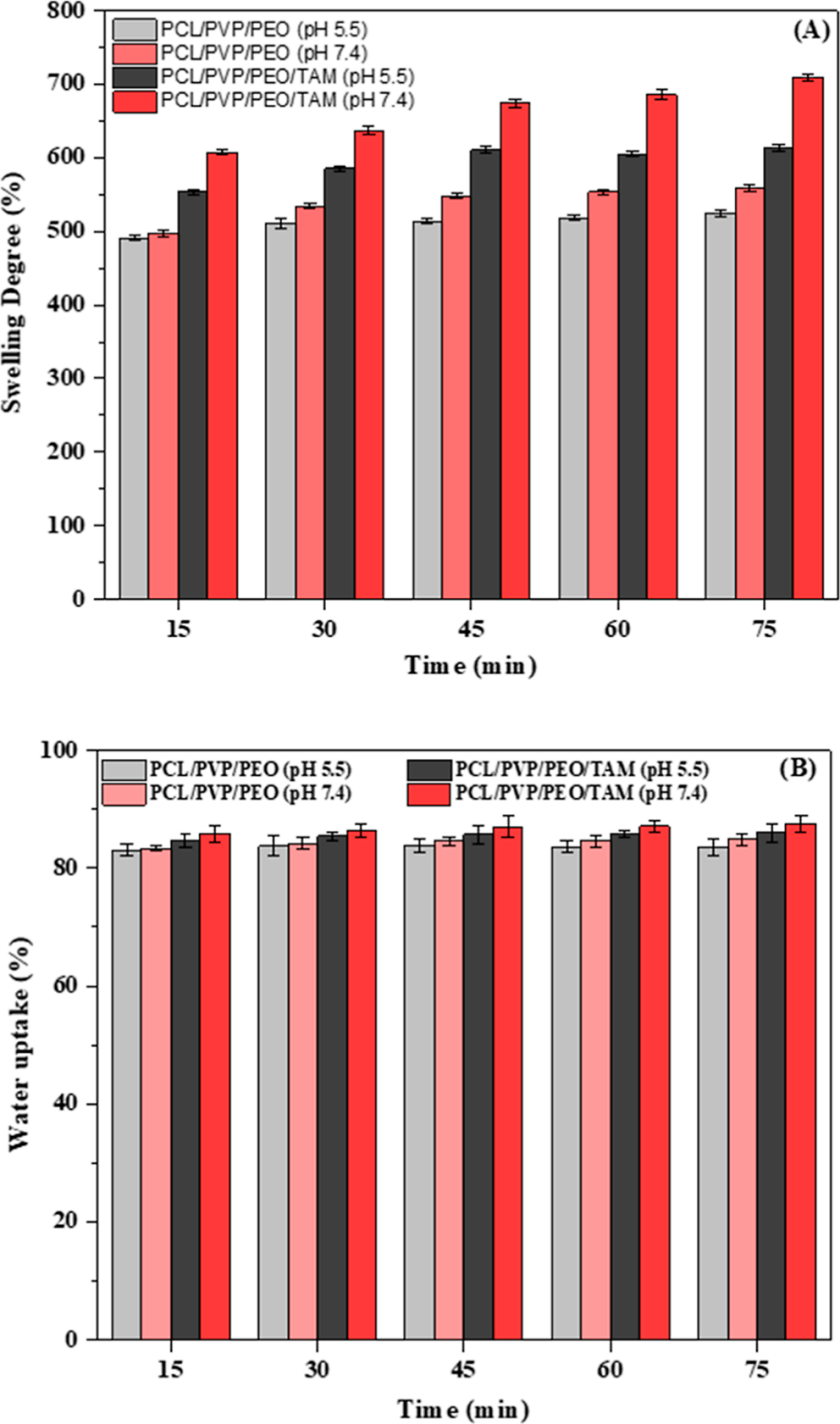
Time-dependent swelling degree (A) and water uptake (B) of PCL/PVP/PEO and TAM-loaded PCL/PVP/PEO fiber mats at pH 5.5 and pH 7.4.

Water uptake measurements further supported the hydration trends observed in the swelling analysis (Figure 8B). All groups exhibited rapid initial water uptake followed by a gradual approach toward plateau values, consistent with typical water absorption behavior of hydrophilic electrospun membranes.^68,69^ At 15 min, water uptake values at pH 5.5 and pH 7.4 were 83.0 ± 0.99% and 83.3 ± 0.45%, respectively; these increased slightly to 83.6 ± 1.34% and 84.8 ± 1.12% by 75 min. For TAM-loaded fibers, uptake at 15 min was 84.6 ± 1.01% (pH 5.5) and 85.8 ± 1.38% (pH 7.4), reaching 86.0 ± 1.10% and 87.5 ± 1.39% at 75 min. The consistently higher water uptake values for TAM-loaded fibers reflect an increased capacity for water retention, further confirming the effect of drug incorporation on the hydrophilic behavior of the nanofibers.^67^ Overall, the combined swelling degree and water uptake results indicate that TAM-loaded PCL/PVP/PEO fibers exhibit enhanced hydration properties compared with drug-free counterparts, particularly under pH 7.4, which may support faster drug diffusion and release under physiological conditions. These findings align with previous studies on pH-responsive and drug-loaded electrospun systems where increased hydrophilicity and swelling capacity correlated with improved drug release performance.^70^

### 3.7 Biodegradation Behavior

Figure 9 presents the *in vitro* hydrolytic degradation profiles of PCL/PVP/PEO and TAM-loaded PCL/PVP/PEO fiber mats incubated in PBS at 37 °C under pH 7.4 and pH 5.5 conditions for 28 days. In all groups, a measurable increase in weight loss was observed with incubation time, indicating limited hydrolytic degradation of the nanofibrous constructs, which is consistent with recent findings on PCL-based electrospun systems in PBS.^71,72^ Notably, the samples incubated at pH 5.5 consistently exhibited slightly higher weight loss compared to those at pH 7.4, indicating that an acidic environment moderately accelerates hydrolytic cleavage of the ester backbone. This pH-dependent behavior of polyester degradation has been documented in several studies, showing accelerated hydrolysis under acidic conditions.^73^ When comparing PCL/PVP/PEO and TAM loaded PCL/PVP/PEO fibers, TAM incorporation resulted in a moderately lower weight loss at both pH values. This observation suggests that TAM presence may be associated with a relatively denser nanofiber structure and reduced water penetration into the polymer matrix. Similar drug-induced modulation of degradation behavior has been reported for other drug-loaded electrospun systems. Overall, the relatively moderate degradation rates observed in both systems align with the well-recognized slow hydrolytic degradation profile of PCL in aqueous media, while the hydrophilic components of the blend contribute to an early water uptake without causing rapid structural disintegration.^71,72^ Furthermore, the relatively low weight loss values (∼5–7% after 28 days) indicate that the developed nanofibrous systems are slowly degradable under the investigated conditions.

**Figure 9:**
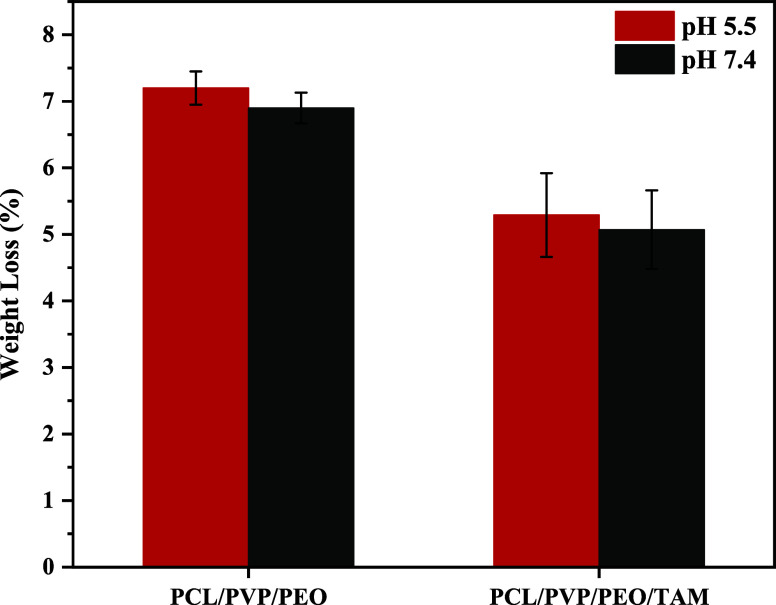
*In vitro* hydrolytic degradation (weight loss) of PCL/PVP/PEO and TAM-loaded PCL/PVP/PEO fiber mats in PBS at pH 7.4 and pH 5.5 (37 °C, 28 days).

### 3.8 *In Vitro* Drug Release

TAM was loaded onto the fiber mats with approximately 99% loading efficiency. The reasons for the maximum loading efficiency are (i) no drug loss occurs during the production of the fiber mats because of the electrospinning technique, and (ii) hydrogen bonds and coordination interactions occur.^5^ This finding is consistent with previous studies of drugs loaded onto fibers. For instance, Jaisankar et al. loaded 5-fluorouracil drug on MgO/PCL nanofiber mats and the mats displayed around 98–99% of drug loading efficiency.^5^

Electrospun nanofiber mats are promising carriers for *in vitro* drug delivery due to their high surface-to-volume ratios and flexibility. Unlike systemic nanocarrier formulations, the current electrospun fiber mat system was proposed as a macroscopic local release platform designed for localized application, such as implantation or postoperative placement into the tumor site. This local application approach aimed to minimize systemic exposure to TAM and associated side effects while also enabling sustained release of TAM directly into the target tissue. Furthermore, the pH-sensitive release behavior may further enhance efficacy through selectively accelerated release of the drug in cancerous tissues. Therefore, TAM release profile was investigated at pH 5.5 and 7.4 (37 °C). A neutral pH of 7.4 was chosen to simulate blood and plasma conditions, representing the environment encountered during intravenous administration, while an acidic pH of 5.5 was selected to mimic the tumor microenvironment, which is more acidic than normal tissues.^74^ Figure 10 shows the cumulative TAM release curves from PCL/PVP/PEO mats at different pH values. In general, our results revealed that TAM exhibited very different release rates from PCL/PVP/PEO fibers at different pH values. This demonstrated that the designed carrier system had pH-sensitive release properties. Moreover, because TAM is an acid-sensitive drug, the drug release was affected by the pH value. As expected, higher TAM release was observed at pH 5.5, the cumulative release value reached 100% after 100 h. At a neutral pH, a drug slower release was appeared. At pH 7.4, a 36% release was observed after 300 h of the experiment. However, after this time, the cumulative release values were determined to be 43% and the release stabilized after 350 h of the experiment. The fiber mat system produced by the electrospinning method was stabilized primarily through noncovalent interactions, including hydrogen bonds, dipole–dipole interactions, and hydrophobic interactions between polymer chains and TAM molecules. These interactions are shown in Scheme 1. PVP and PEO contain polar functional groups that can interact with TAM via hydrogen bonds and dipole interactions, while the hydrophobic PCL phase contributes to sustained drug retention and delayed diffusion. The immediate release phase refers to the rapid release of TAM present on the fiber surfaces. Subsequently, TAM present within the fibers gradually diffuses to the fiber surface and then into the medium solution, resulting in a slow diffusion rate.^75^ These results indicated that TAM release from PCL/PVP/PEO mats is pH-dependent and that the amount of TAM release increases as the environment becomes more acidic. Although swelling is higher at pH 7.4, the faster drug release at pH 5.5 may be due to weakened intermolecular interactions and increased drug mobility under acidic conditions. Overall, the release of TAM shows that the pH-sensitive fiber could be a suitably controlled delivery platform for breast cancer treatment. The pH-sensitive release behavior can also be associated with the presence of hydrophilic PVP and PEO within the nanofibrous matrix. Both polymers possess strong water affinity and promote rapid water penetration, swelling, and polymer chain relaxation in aqueous environments. As supported by the swelling and water uptake results (Figure 8), the incorporation of PVP and PEO enhanced the hydration behavior of the fiber mats, facilitating diffusion of the release medium into the polymer network. These hydrophilic interactions may accelerate TAM diffusion from the nanofibers, particularly under acidic conditions, where increased polymer–medium interactions and structural relaxation can contribute to faster drug release behavior. Additionally, the variation observed in the drug release profile may be attributed to pH-dependent changes in the solubility and ionization behavior of TAM. Similar effects of hydrophilic polymeric components on swelling-assisted and pH-responsive drug release behavior have been reported in electrospun drug delivery systems.^63,76,77^

**Figure 10:**
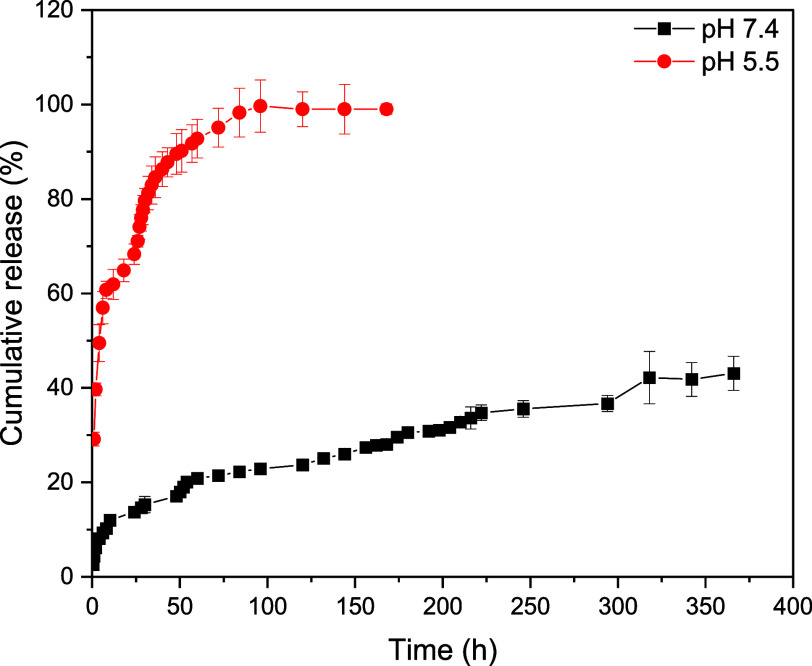
Release profile of TAM from fiber in two different buffer media (pH 5.5 and 7.4).

To understand the mechanism of TAM release, the drug release data were fitted onto different kinetic models and the curves obtained are shown in Figure 11. In addition, corresponding expressions and *R* ^2^ values are tabulated in Table 1. The higher the *R* ^2^ value of the kinetic model, the more closely the oscillation curve fits the kinetic model.^75^ The models that best fit the experimental data are shown in bold. By analyzing TAM release data at pH 5.5, it is observed that Hixson–Crowell model was the one that best fits the experimental data. *R* ^2^ value for the Hixson–Crowell model was found to be 0.9857. Conversely, at pH 7.4, the Higuchi model produced a higher *R* ^2^ value (0.9876). On the other hand, the most commonly used kinetic model for investigating the kinetics of drug release from polymeric matrices is the Korsmeyer–Peppas model. It assumes that polymer erosion, as well as Fickian diffusion, plays a role in drug release from the carrier system. The mechanism associated with drug release is represented by the value “*n*”. If the “*n*” value is less than 0.5, drug release is regulated by Fickian diffusion; if it is greater than 0.5, it is controlled by non-Fickian (abnormal) diffusion.^78^ In the present study, the “*n*” values for all pH values ranged from 0.248 to 0.378, following the Fickian diffusion model. Furthermore, high *R* ^2^ values of 0.9700 and 0.9863 were obtained in the Korsmeyer–Peppas model for pH 5.5 and 7.4, respectively. Therefore, analysis of the relevant parameters shows that the main release mechanism of TAM from fiber mats was Fickian diffusion at all pH conditions.

**Figure 11:**
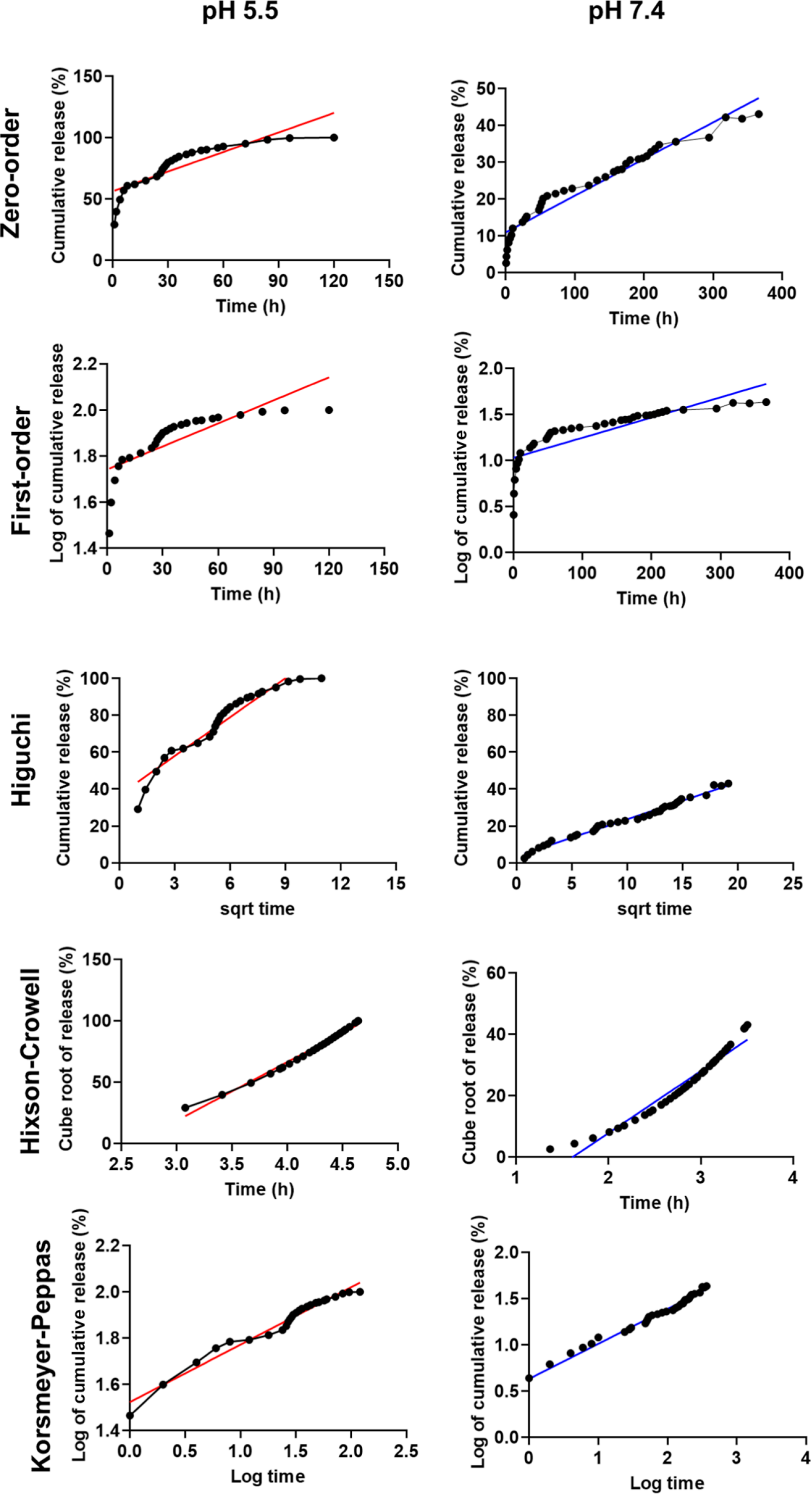
Plots of different types of drug release kinetic modeling.

**Table 1:** Mathematic Modeling and TAM Release Kinetics from Fibers

	zero order		first order		Higuchi		Hixson–Crowell		Korsmeyer–Peppas		
pH	*R* ^2^	*k*_0_	*R* ^2^	*k*_1_	*R* ^2^	*k*_H_	*R* ^2^	*k*_HC_	*R* ^2^	*n*	*k*_P_
5.5	0.7320	0.5319	0.5967	0.0076	0.9135	7.02	**0.9857**	47.586	0.9700	0.248	33.3
7.4	0.9325	0.0996	0.6784	0.0051	**0.9876**	2.01	0.9497	20.233	0.9863	0.378	4.28

### 3.9 Biocompatibility and *In Vitro* Anticancer Activity

The fiber mats were treated with fibroblast L929 and MCF-7 cells by the MTT assay to determine the biocompatibility and cytotoxic effects and the observed results are given in Figure 12. Additionally, free TAM was evaluated as a control group under the same experimental conditions. However, at the concentration used in this study, free TAM caused almost complete loss of viability in both L929 and MCF-7 cells, preventing a meaningful comparison in terms of selective cytotoxicity (data not shown). Figure 12 shows the cell viability results of healthy L929 cells treated with PCL/PVP/PEO and TAM-loaded PCL/PVP/PEO fiber mats for 24, 48, and 72 h. PCL/PVP/PEO fiber samples showed high cell viability (80.6, 89.3, and 90%) for 24, 48, and 72 h, indicating that PCL/PVP/PEO fibers are biocompatible and exhibit minimal cytotoxic effect on L929 cells. This is likely a result of the inherent biocompatibility and cell proliferation-promoting properties of the polymeric materials used in the fiber matrix. Polymers such as PCL, PVP, and PEO, commonly used in fiber production, have been shown to support cell attachment and proliferation thanks to the significant surface area provided by the fiber matrix, which mimics the extracellular matrix. These materials can act as scaffolds, enhancing cell growth and survival by providing physical support and promoting positive interactions between cells and the material surface.^79^ In contrast, a decrease in viability was observed with TAM loading. TAM-loaded PCL/PVP/PEO fiber mats showed cell viability of 69.7, 88.5, and 63.7% for 24, 48, and 72 h. In particular, viability dropped to approximately 63.7% at 72 h. This indicated that TAM also has some suppressive effect on healthy cells. Furthermore, *in vitro* anticancer effect of PCL/PVP/PEO and TAM-loaded PCL/PVP/PEO fiber mats was evaluated against MCF-7 breast cancer cells for 24, 48, and 72 h., and the results are shown in Figure 12. After 24, 48, and 72 h, the cell viability rates for PCL/PVP/PEO fiber mats were determined to be 83.2%, 81.8%, and 70.5%, respectively, while cell viability rates were calculated as 69.9%, 68.9%, and 57.7%, respectively, for TAM-loaded PCL/PVP/PEO fiber mats. Interestingly, the effect of PCL/PVP/PEO fiber mats on cancer cells was observed. This can be interpreted as the surface properties of the polymeric matrix or the released polymer particles slightly suppressing the aggressive proliferation metabolism of cancer cells. Furthermore, the decrease in viability observed with the application of PCL/PVP/PEO fiber matrices can be attributed to the tendency of nanofibers to adsorb serum proteins and growth factors in the culture medium due to their high surface area/volume ratio. However, the significant difference between TAM-loaded PCL/PVP/PEO fiber mats and the empty group confirmed that the main source of cytotoxicity was not nutrient absorption, but the anticancer effect of the released TAM. Although the difference between blank and TAM-loaded PCL/PVP/PEO fiber mats was moderate, fluorescence microscopy analyses additionally revealed enhanced apoptotic/cytotoxic signals in TAM-loaded PCL/PVP/PEO fiber mats, supporting the MTT findings. This moderate level of cancer activity is due to the hydrophobic nature of the fiber mat, the limited cell adhesion properties of the cell–fiber mat contact, and the slow hydrolysis of hydrogen bonds. Therefore, TAM drug could not be effectively released from the fiber mat.^80^ These results were consistent with *in vitro* drug release studies. Overall, these results clearly confirmed that fiber mats exhibited a favorable safety profile and selective cytotoxicity against cancer cells. Therefore, the fiber mats would be a biocompatible scaffold in the field of drug delivery for cancer therapy after careful dose optimization.

**Figure 12:**
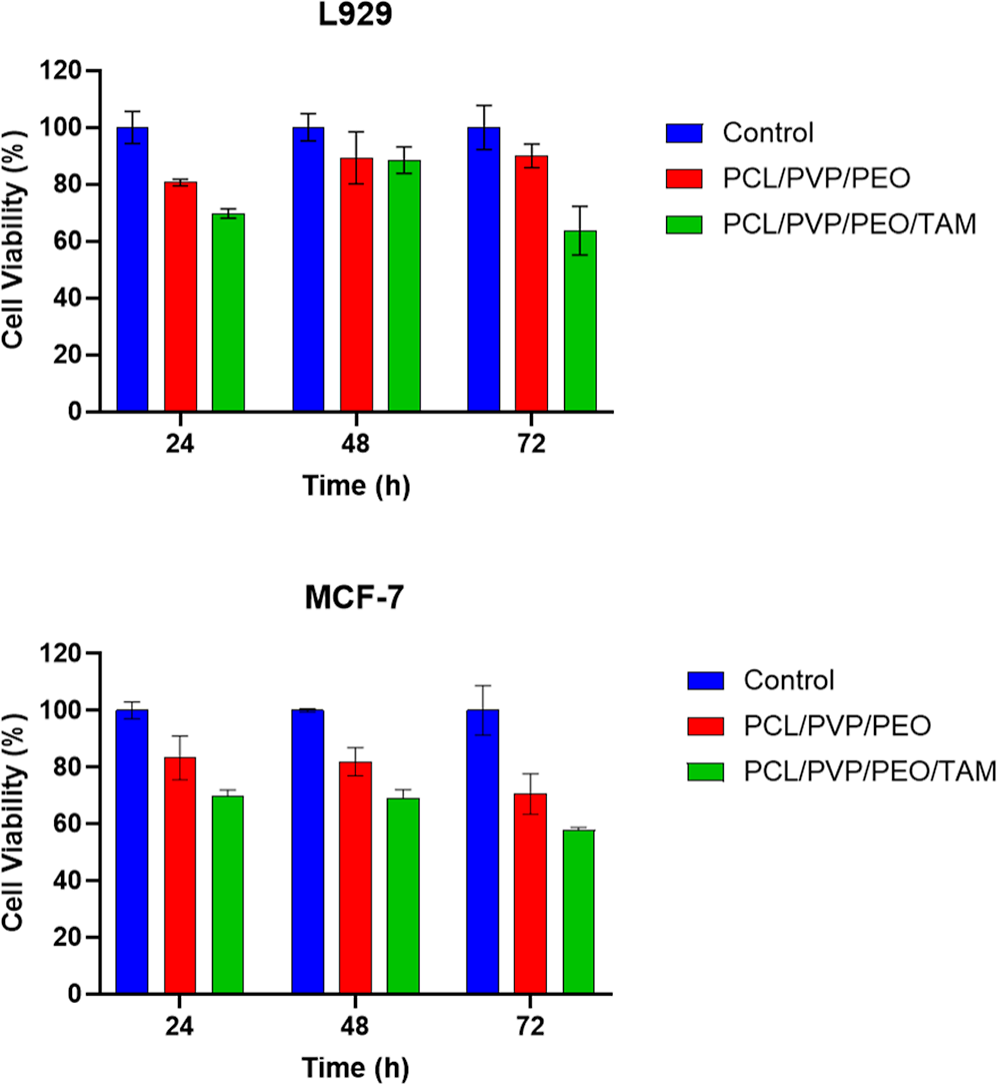
Cellular viability (using MTT assay) in the L929 and MCF-7 cell cultures on the treatment of blank, PCL/PVP/PEO, and TAM-loaded PCL/PVP/PEO fiber mats for 24, 48, and 72 h.

Figure 13 shows quantitative fluorescence intensity analysis of apoptotic/cytotoxic responses in L929 and MCF-7 cells treated with control, PCL/PVP/PEO, and TAM-loaded PCL/PVP/PEO electrospun fibers after 24, 48, and 72 h of incubation. According to AO/EtBr fluorescent staining results, no apoptotic signal was observed in either MCF-7 or L929 cells in the control groups. While a low level of apoptotic effect was determined in the PCL/PVP/PEO group, significant apoptotic activity was detected in the PCL/PVP/PEO/TAM group, especially in MCF-7 cells. The apoptotic signal rate in MCF-7 cells was calculated as 66.67% at 24 h, 68.83% at 48 h, and 50.44% at 72 h. In L929 cells, the apoptotic signal rates in the same group were found to be 49.05%, 18.06%, and 30.94%, for 24, 48, and 72 h, respectively. The obtained data indicated that the PCL/PVP/PEO/TAM fiber mats produced a higher apoptotic effect, especially in MCF-7 breast cancer cells.

**Figure 13:**
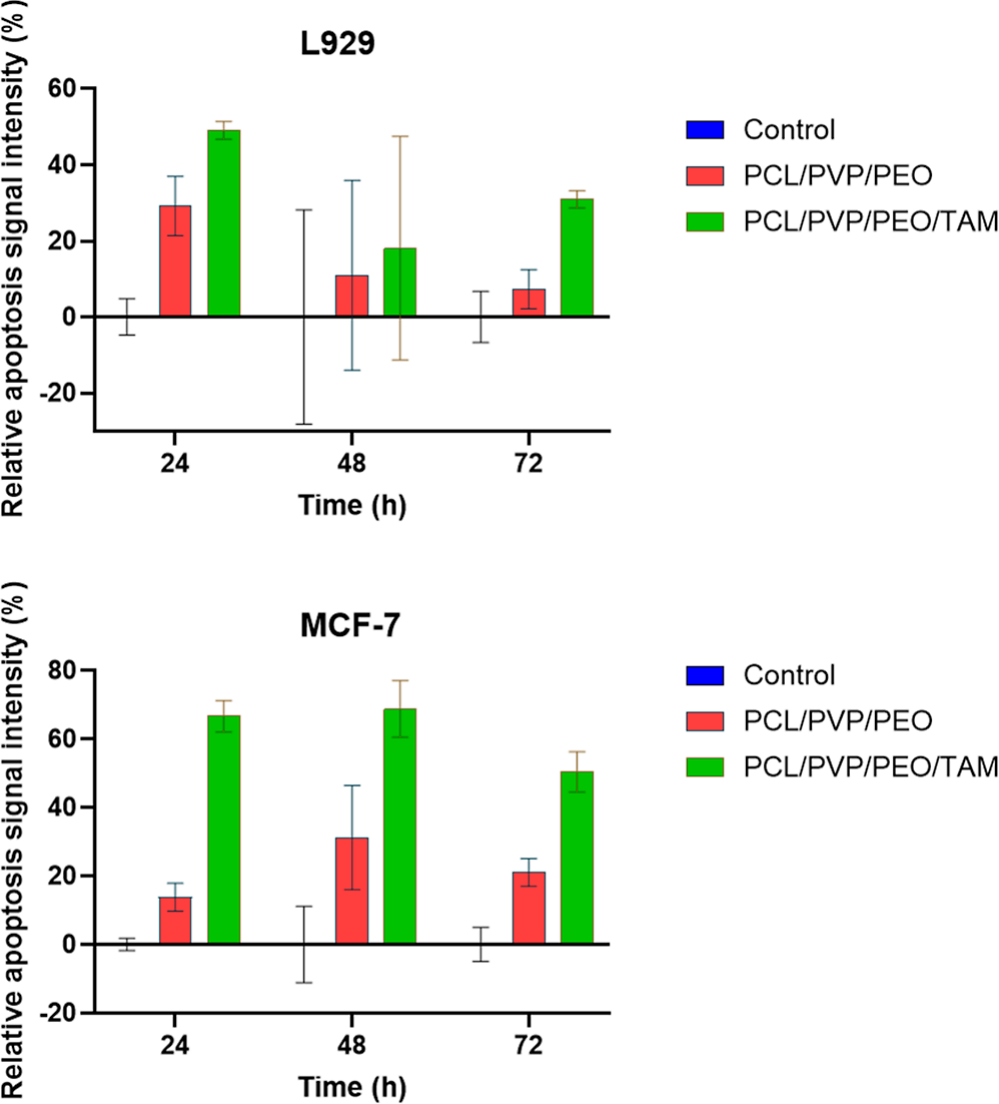
Quantitative fluorescence intensity analysis of apoptotic/cytotoxic responses in L929 and MCF-7 cells treated with control, PCL/PVP/PEO, and TAM-loaded PCL/PVP/PEO electrospun fibers after 24, 48, and 72 h of incubation. Data represent mean ± standard deviation.

Figure 14 indicates fluorescence microscopy images showing apoptotic/cytotoxic responses of L929 and MCF-7 cells cultured with control, PCL/PVP/PEO, and TAM-loaded PCL/PVP/PEO electrospun fibers after 24, 48, and 72 h of incubation. When AO/EtBr fluorescence images were examined, green fluorescence was predominantly observed in both L929 and MCF-7 cells in the control groups, and it was determined that cell viability was preserved. In the PCL/PVP/PEO group, increasing orange/red fluorescence signals over time were particularly noticeable in MCF-7 cells. The most significant change was observed in the PCL/PVP/PEO/TAM group; the intense orange-red fluorescence formation at 24, 48, and 72 h, especially in MCF-7 cells, indicated increased apoptotic cell death. In contrast, the preservation of more dominant green fluorescence in L929 cells supports the idea that electrospun PCL/PVP/PEO/TAM fiber mats exerted a more selective anticancer effect on cancer cells compared to normal fibroblast cells. When evaluated together with the MTT cell viability results, the increase in apoptotic signal accompanying the decrease in viability in MCF-7 cells indicated that the anticancer efficacy of the developed system was related to the apoptotic mechanism.

**Figure 14:**
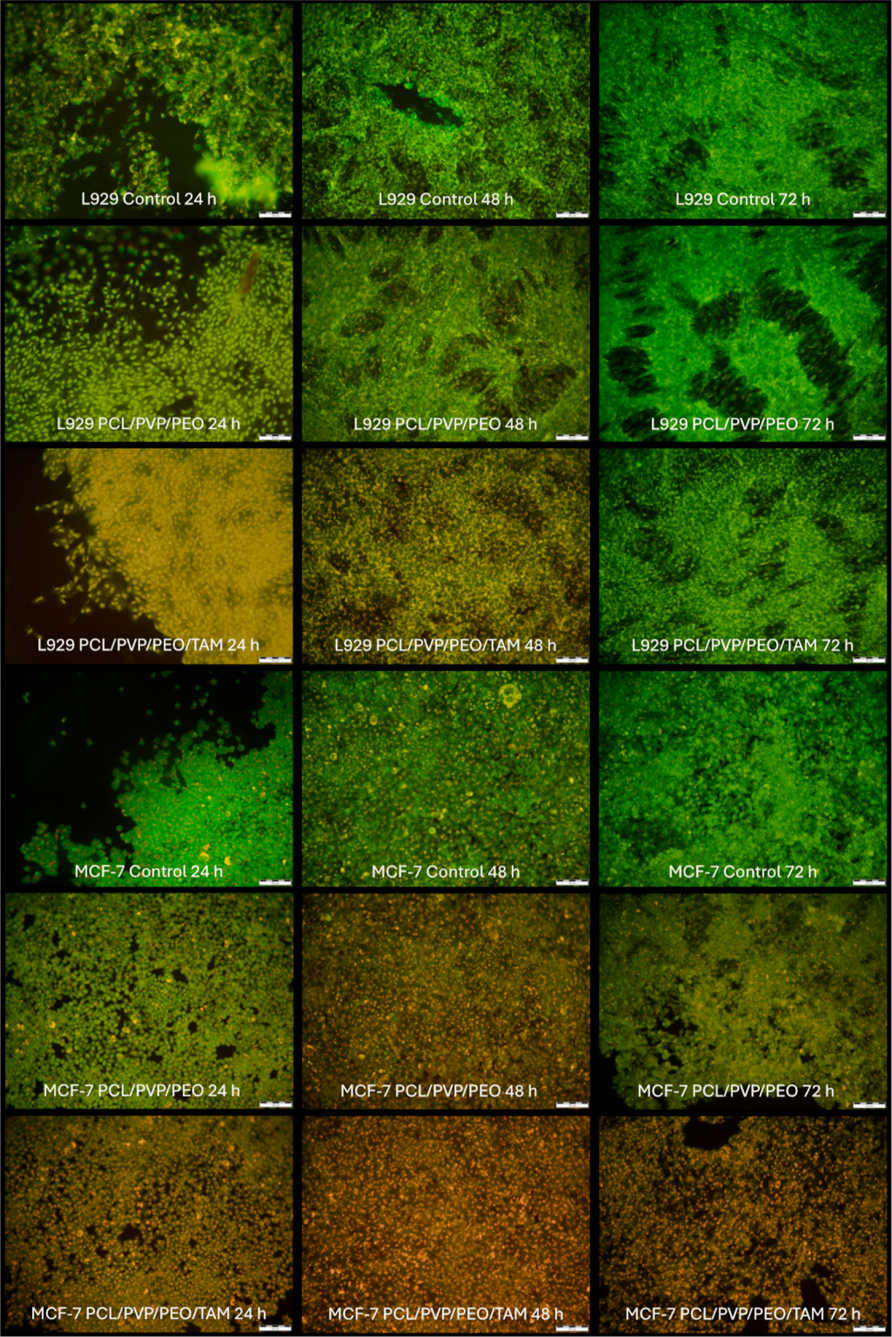
Fluorescence microscopy images showing apoptotic/cytotoxic responses of L929 and MCF-7 cells cultured with control, PCL/PVP/PEO, and TAM-loaded PCL/PVP/PEO electrospun fibers after 24, 48, and 72 h of incubation.

## 4 Conclusions

In short, our study demonstrated the ability to load a poorly water-soluble drug (i.e., TAM) into electrospun PCL/PVP/PEO fiber mats of various compositions. The fiber mats were subjected to thorough characterization through various analytical methods, including FTIR, XRD, SEM, TGA, and EDS. FTIR analysis confirmed that TAM drug was successfully incorporated into the produced fibers. SEM analysis showed that the pH-controlled PCL/PVP/PEO fiber mats were bead-free, randomly aligned, and had a continuous rod-shaped structure with the average range of 346–418 nm. In contrast to the neutral physiological pH of 7.4, the pH-sensitive fiber mats obtained demonstrated increased drug diffusion under acidic conditions (pH 5.5) resembling the tumor microenvironment. According to the *in vitro* release analysis, the cumulative released TAM in the first 24 h was about 65% at pH 5.5, while at pH 7.4, the release percentage was under 15%. The kinetic analysis revealed that the drug release mechanism adopted different models, including Higuchi, Hixson–Crowell, and Korsmeyer–Peppas kinetics. Most importantly, the MTT assay results confirmed fiber mats were cytotoxic to the breast cancer cell line, MCF-7, than pristine fibers. The fluorescent staining images revealed that more apoptotic cells appeared when cancer cells are exposed with fiber mats. Overall, these findings suggested that the current electrospun fiber mat system was proposed as a macroscopic local release platform designed for localized application, such as implantation or postoperative placement in the tumor site. Our next step is to conduct in vivo studies to confirm the expected improvements in the bioavailability and overall stability of TAM embedded in microfibers. Thus, this study will facilitate the targeted delivery of TAM to tumor tissues.
